# Synthesis, Structural Characterization, and Antibacterial Activity of Novel Erbium(III) Complex Containing Antimony

**DOI:** 10.1155/2018/4313197

**Published:** 2018-03-20

**Authors:** Ting Liu, Rong-Gui Yang, Guo-Qing Zhong

**Affiliations:** School of Material Science and Engineering, Southwest University of Science and Technology, Mianyang 621010, China

## Abstract

The novel 3D edta-linked heterometallic complex [Sb_2_Er(edta)_2_(H_2_O)_4_]NO_3_·4H_2_O (H_4_edta = ethylenediaminetetraacetic acid) was synthesized and characterized by elemental analyses, single-crystal X-ray diffraction, powder X-ray diffraction (XRD), Fourier transform infrared spectroscopy (FTIR), and thermal analysis. The complex crystallizes in the monoclinic system with space group *Pm*. In the complex, each erbium(III) ion is connected with antimony(III) ions bridging by four carboxylic oxygen atoms, and in each [Sb(edta)]^−^ anion, the antimony(III) ion is hexacoordinated by two nitrogen atoms and four oxygen atoms from the edta^4−^ ions, together with a lone electron pair at the equatorial position. The erbium(III) ion is octacoordinated by four oxygen atoms from four different edta^4−^ ions and four oxygen atoms from the coordinated water molecules. The carboxylate bridges between antimony and erbium atoms form a planar array, parallel to the (1 0 0) plane. There is an obvious weak interaction between antimony atom and oxygen atom of the carboxyl group from the adjacent layer. The degradation of the complex proceeds in several steps and the water molecules and ligands are successively emitted, and the residues of the thermal decomposition are antimonous oxide and erbium(III) oxide. The complex was evaluated for its antimicrobial activities by agar diffusion method, and it has good activities against the test bacterial organisms.

## 1. Introduction

Much attention is currently focused on the rational design and controlled synthesis of metal-organic complexes with novel topological structure because of various potential applications of these complexes as function materials, catalysts, and medicaments [1–5]. Metal-based drugs continue to play a very important role in clinical medicine, and antimony-based metallotherapeutic drugs were used in medical applications very early in the past. Nowadays many of antimony(III) complexes have been clinically used because of their biological activities and drug efficacies [6–19], such as the treatment of a variety of microbial infections including leishmaniasis, parasitic diseases, diarrhea, peptic ulcers, helicobacter pylori, and so forth. More recently, the use of antimony complexes in cancer chemotherapy has become a topic of interest, and antimony(III) compounds have been tested *in vitro* for their cytotoxic effects on the proliferation of some leukemia and solid tumor cells [20–26].

The aminopolycarboxylate ligands can act as multidentate ligand, and their important characteristic bases on the bridging mode of the carboxylate groups [27–31]. Among the investigation of syntheses and structures of various aminopolycarboxylate complexes, heterometallic complexes are of great interest in view of their fascinating structural diversity and potential applications. Some edta-linked heterometallic complexes containing transition metals have been synthesized and structurally characterized (H_4_edta = ethylenediaminetetraacetic acid) [32, 33]. However, less work on the main group elements participating in the heterometallic complexes due to the particularities of main group elements has been reported [34–40]. Antimony compounds are easy to be hydrolyzed in aqueous solutions, which makes difficult to synthesize their complexes [23], so the study of antimony complexes is much less than that of transition metal and rare earth metal complexes.

In continuation of our interest on the antimony(III) [41–43] and bismuth(III) [44, 45] complexes with aminopolycarboxylate ligands, we report herein a novel antimony-based heterometallic complex [Sb_2_(edta)_2_-*μ*_4_-Er(H_2_O)_4_]NO_3_·4H_2_O; its composition and crystal structure have been characterized by elemental analyses, FTIR spectrum, single crystal X-ray diffraction, and thermal analysis. The complex has been evaluated for its antimicrobial activities by agar diffusion method. The synthesis method for the complexes of the antimony-transition metal and antimony-lanthanide with aminopolycarboxylic acid ligands is different. Significant knowledge about these complexes is very interesting due to their fascinatingly special structures and interesting properties, and antimony ion has weaker coordination ability than transition metal or lanthanide series ions leading to fewer reports about its complexes. The structural variety of antimony complexes is not similar to bismuth complexes. Bismuth(III) displays a marked propensity to form the complexes with high coordination number, such as the coordination number of 6–10 [32]. However, antimony(III) is generally hexacoordinated, and the stereochemistry of antimony(III) complexes is usually based on a distorted trigonal bipyramid with a pair of active lone electrons in one of the trigonal planar sites. The lone pair electrons located on antimony atom plays an important role in the final geometry obtained [23].

## 2. Experimental Section

### 2.1. Materials and Physical Measurements

All chemicals purchased in the experiments were of analytical reagent and used as received without further purification, and the solvents were also commercially available and further purified before use. The antimony trichloride, erbium nitrate hexahydrate, ethylenediaminetetraacetic acid, and ammonium bicarbonate were purchased from Sinopharm Chemical Reagent Co. Ltd. of Shanghai. The complex [Sb(Hedta)]·2H_2_O was synthesized as described in the literature [46]. *Staphylococcus aureus*, *Escherichia coli*, *Salmonella typhi*, *Bacillus subtilis*, and *Staphylococcus epidermidis* were provided by the 404 hospital of Sichuan Mianyang.

Elemental analyses of C, H, and N were performed on an elemental analysis service of vario EL III elemental analyzer. Melting point was determined in capillary tubes on an X4 melting point apparatus. Molar conductance was measured by a DDS-11A conductometer. XRD pattern was recorded on a D/max-II X-ray diffractometer in the diffraction angle range of 5–80°. FTIR spectrum was measured with a KBr disk on a Nicolet 570 FT-IR system. Thermal gravimetric (TG) analysis was carried out on a STA 449C differential thermal balance in air, with a heating rate of 10°C·min^−1^ and *α*-Al_2_O_3_ reference.

### 2.2. Synthesis of [Sb_2_Er(edta)_2_(H_2_O)_4_]NO_3_·4H_2_O

2 mmol (0.90 g) of [Sb(Hedta)]·2H_2_O was dissolved in 60 mL hot distilled water, and the solution was heated to 95°C. Then, 2 mmol (0.16 g) NH_4_HCO_3_ was gradually added to the above solution, and the solution was stirred for about 30 min. After cooling the solution to room temperature, 2 mmol (0.92 g) Er(NO_3_)_3_·6H_2_O was added to the above solution; in this case, the transparent solution was obtained. The mixture solution was held for a week, and the pink block crystals were isolated from the solution. The yield was about 58%. m.p.: 192°C (decomposition). Anal. Calc. for the complex C_20_H_40_N_5_O_27_ErSb_2_: C, 20.13; H, 3.38; N, 5.87%. Found: C, 20.01; H, 3.22; N, 5.51%. FTIR (KBr disk): 3426(s), 2986(w), 2956(w), 1654(s), 1593(s), 1508(w), 1469(m), 1448(m), 1402(w), 1385(m), 1356(m), 1317(m), 1294(m), 1254(m), 1158(m), 1082(m), 1039(m), 1000(m), 948(m), 916(m), 864(m), 828(m), 741(w), 710(m), 661(m), 619(w), 594(w), 562(m), 529(m), 516(w), 460(m), 448(m), 434(m), 426(m), 420(m), and 407(m) cm^−1^.

### 2.3. X-Ray Cystallography

All measurements were made on a Siemens P4 diffractometer at 289(2) K using graphite monochromated Mo K_*α*_ (*λ* = 0.71073 Å). A pink block with dimensions 0.48 × 0.44 × 0.20 mm^3^ was mounted on a glass fiber. Diffraction data were collected in *ω* mode in the range 1.84° < *θ* < 26.00°. Data were corrected for Lorentz and polarization effects, and an empirical absorption correction was applied. The structures were solved by the SHELXS-97 program and refined using full-matrix least squares on *F*^2^ with the SHELXL-97 program [47]. For the complex, the hydrogen atoms attached to the oxygen atoms of water molecules were not located from the difference Fourier map due to the effect of heavy erbium and antimony atoms, while other nonhydrogen atoms were refined anisotropically, and hydrogen atoms were introduced at the calculated positions. CCDC 637089 contains the supplementary crystallographic data for the title complex. These data can be obtained free of charge via http://www.ccdc.cam.ac.uk/conts/retrieving.html or from the Cambridge Crystallographic Data Centre, 12 Union Road, Cambridge CB2 1EZ, UK; fax: (+44) 1223-336-033 or e-mail: deposit@ccdc.cam.ac.uk.

## 3. Results and Discussion

The complex is stable in air and soluble in hot water and difficult to dissolve in most common organic solvents and slightly soluble in DMF. The molar conductance values of the complex in DMF and deionized water (10^−3^ mol·L^−1^ solution at 25°C) are 88.2 and 92.5 S·cm^2^·mol^−1^, respectively. The results show that the complex belongs to 1 : 1 electrolyte nature [48].

### 3.1. Crystal Structure Analysis

**Figure 1:**
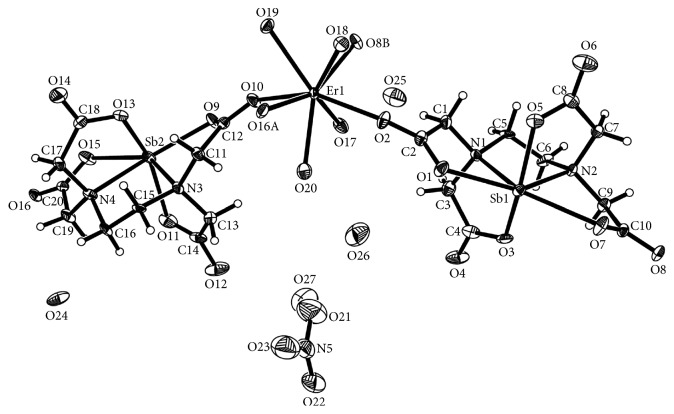
Thermal ellipsoid representation (at 50% probability) of molecular structure unit of the complex. All the H atoms are omitted for clarity. Symmetry codes: A *x* − 1/2, −*y*, *z* − 1/2; B *x* + 1/2, −*y* + 1, *z* − 1/2.

**Figure 2:**
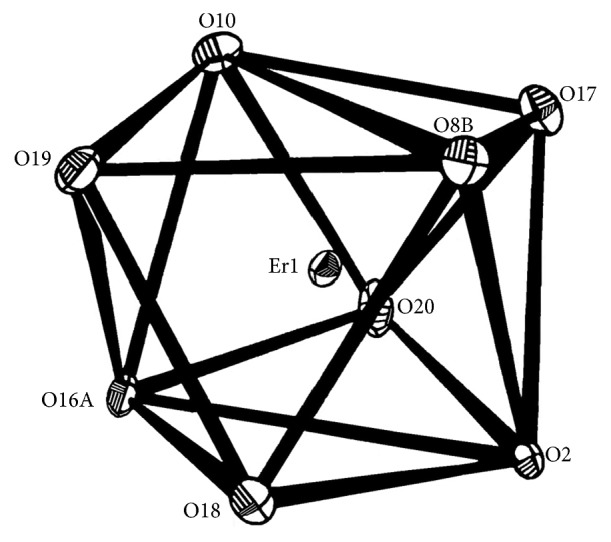
Coordination polyhedron structure of Er(III).

The molecular structure of the title complex with atomic labeling scheme is shown in Figure 1. Crystallographic data and structure refinement parameters of the complex are given in Table 1, and selected bond lengths and bond angles are given in Table 2. The asymmetric unit of the complex consists of a crystallographically independent heterometallic motif [Sb_2_-*μ*_4_-(edta)_2_Er(H_2_O)_4_]^+^, nitrate counterion, and four free water molecules. Each edta^4−^ ion consists of carboxylate groups adopting monodentate mode coordination to the antimony ion and bidentate bridging over one erbium ion and two different antimony ions. Each erbium(III) ion has a distorted trigonal dodecahedron environment with an O_8_ donor atom array: four bridged oxygen atoms [*µ*-O(2), *µ*-O(10), *µ*-O(8B), and *µ*-O(16A)] from four edta^4−^ ligands with Er–O bond distances ranging from 2.285(7) to 2.298(7) Å, four oxygen atoms from the four coordinated water molecules with Er–O bond distances ranging from 2.347(9) to 2.432(7) Å, and the coordination structure of the erbium(III) is shown in Figure 2. The antimony(III) ion is hexacoordinated by four oxygen atoms and two nitrogen atoms from the edta^4−^ ligand, and the lone pair electrons on the antimony atom cause the coordination geometry to be distorted octahedron with two oxygen atoms [O(3) and O(5)] at the axial sites. Two oxygen [O(1) and O(7)] and two nitrogen [N(1) and N(2)] atoms occupy the equatorial plane. The sum of the equatorial bond angles O(1)–Sb(1)–O(7), N(1)–Sb(1)–N(2), N(1)–Sb(1)–O(1), and N(2)–Sb(1)–O(7) is 361°, which shows that the O(1), O(7), N(1), N(2), and Sb atoms are almost located at one plane. The bond angle O(3)–Sb(1)–O(5) of 146.1° is almost twice as large as the bond angle N(1)–Sb(1)–N(2) (77.0°) or N(2)–Sb(1)–O(7) (67.0°), which may be due to the existence of a lone pair of electron in the diad direction [49]. The distances of the Sb–O bonds are in the range of 2.127(8) to 2.541(8) Å, while the Sb–N bond lengths are in the range of 2.315(8) to 2.332(8) Å. The distances of the bidentate chelating bonds Sb(1)–O(1) (2.459 Å) and Sb(1)–O(7) (2.564 Å) are longer than the monodentate bond distances [Sb(1)–O(3) (2.215 Å) and Sb(1)–O(5) (2.127 Å)]. The O–Sb–O bond angles lie between 80.4(3)° and 148.3(3)°. These bonds and angles are slightly longer and wider, respectively, than those in the complex [CaSb_2_(edta)(H_2_O)_8_]_*n*_ [49]. These bond distances and angles (Table 2) are consistent with those of other edta-Sb compounds [50].

**Figure 3:**
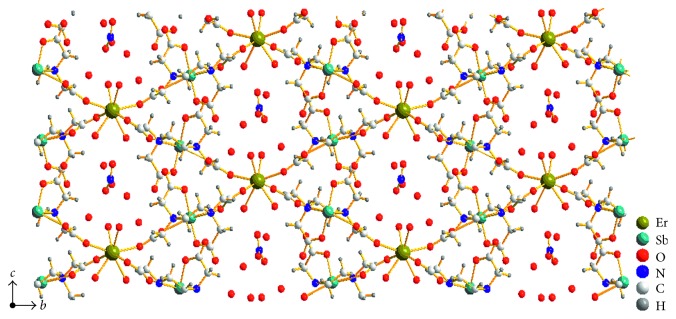
A view of the two-dimensional layer of the complex parallel to the (1 0 0) plane.

**Figure 4:**
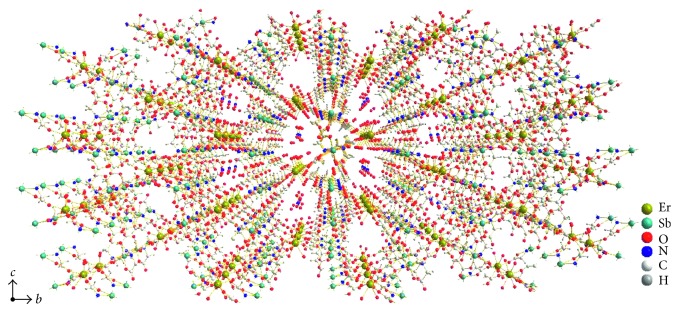
Packing diagram of the title complex viewed along the *a* axis.

The carboxylate bridges between antimony and erbium atoms [O(l)–C(2)–O(2) and O(7)–C(10)–O(8)] form a planar array of metal atoms, with a maximum deviation of 0.416 Å, parallel to the (1 0 0) plane (Figure 3). Furthermore, there is an obvious weak interaction between antimony atom and oxygen atom of the carboxyl group from the adjacent layer. The interaction makes the layers extend to an infinite three-dimensional framework (Figure 4). Hydrogen bonds and short van der Waals force contact between oxygen atoms from carboxyl groups and water molecules and also between water molecules strengthen this three-dimensional arrangement.

### 3.2. FTIR Spectrum

**Figure 5:**
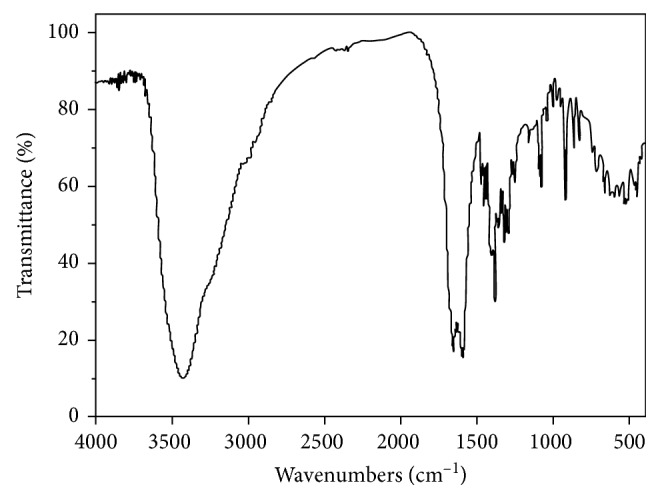
FTIR spectrum of the title complex.

The FTIR spectrum of the title complex is shown in Figure 5. The broad band at about 3426 cm^−1^ is due to *ν*(OH) vibration of the water molecule. The frequency of the peak is higher than 3400 cm^−1^ showing that the oxygen atoms of the water molecule are coordinated to the metal ions [51]. The absorption peaks at 1593, 1402, and 1385 cm^−1^ may be from the asymmetric and symmetric stretching vibration in the carboxyl groups, respectively [52]. It is found that the absorption peak *ν*_as_(COO^−^) at 1690 cm^−1^ of Na_2_H_2_edta is shifted red to 1593 cm^−1^ and the absorption peak *ν*_s_(COO^−^) at 1353 cm^−1^ of Na_2_H_2_edta is shifted blue to 1402 and 1385 cm^−1^ in the complex. The difference values [Δ*ν*(*ν*_as_ − *ν*_*s*_) = 191 and 208 cm^−1^] between the frequencies of the asymmetric and symmetric stretching vibration confirm that the oxygen atoms of carboxylic groups are coordinated to metallic ions by the monodentate mode and bidentate bridge mode in the complex [42], and it is in agreement with the crystal structure. The weaker absorption peaks at about 1356 and 828 cm^−1^ may be from the stretching vibrations in the free nitrate ion. This indicates that the nitrate ion is not coordinated to the metallic ions. The absorption peaks at 1082 and 1039 cm^−1^ may be from various stretching vibrations of the C–N and C–C bonds in the edta^4−^ ligand, respectively. In the far-infrared region, the frequency of the stretching vibration of the Sb–N bonds is 460 and 448 cm^−1^, the frequency of the stretching vibration of the Sb–O bonds is 434 and 426 cm^−1^, respectively. It may be reasonable to assign the peaks at 420 and 407 cm^−1^ to the stretching vibration of the Er–O bonds in the complex [31, 50].

### 3.3. Thermal Analysis

**Figure 6:**
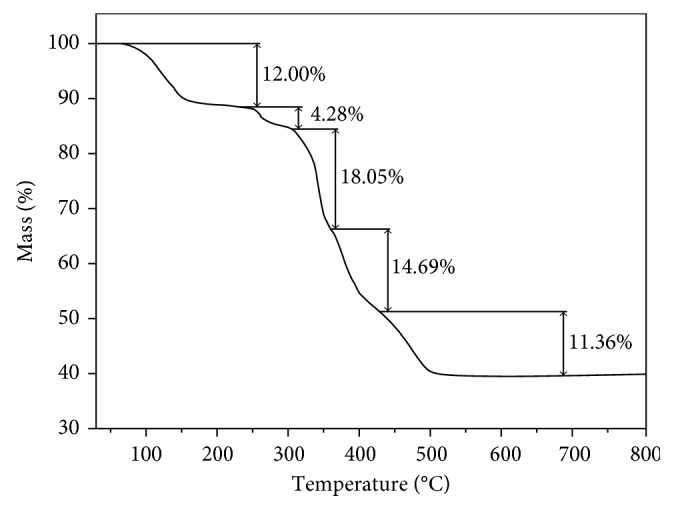
TG curve of the title complex.

Studying the thermal decomposition process of complexes is helpful to the understanding of the coordination structure of these complexes [30, 43]. The TG curve of the complex in air atmosphere from room temperature to 800°C is shown in Figure 6, and the data of possible thermal decomposition processes are listed in Table 3. The first mass loss of 12.00% occurs between 70 and 220°C, corresponding to the gradual loss of the free water molecules and the coordinated water molecules (calculated as 12.08% for 8H_2_O). Then, the sample will gradually lose the free nitrate ion at between 220 and 310°C and the corresponding mass loss of 4.28% (calculated as 4.53%). Between 310 and 360°C, two (CH_2_)_2_NCH_2_COO groups in the complex are oxidized and decomposed, and meanwhile, one quarter oxygen molecules are lost, and the experimental mass loss (18.05%) is close to the calculated one (17.45%). The fourth step mass loss of the complex from 360 to 430°C is 14.69%, corresponding to the mass loss of two N(CH_2_)_3_ groups and two CO molecules (calculated as 14.09%) [30]. Upon further heating, the complex is decomposed completely between 430 and 510°C, and the mass loss of 11.36% in TG curve corresponds to lose the group of four CO molecules and three-fourths of oxygen molecules (calculated as 11.40%). The remaining mass is almost constant until 510°C, and the final residues of the thermal decomposition of the complex are the mixture of Sb_2_O_3_ and Er_2_O_3_, and the experimental result (39.62%) is in agreement with the result of theoretical calculation (40.45%).

**Figure 7:**
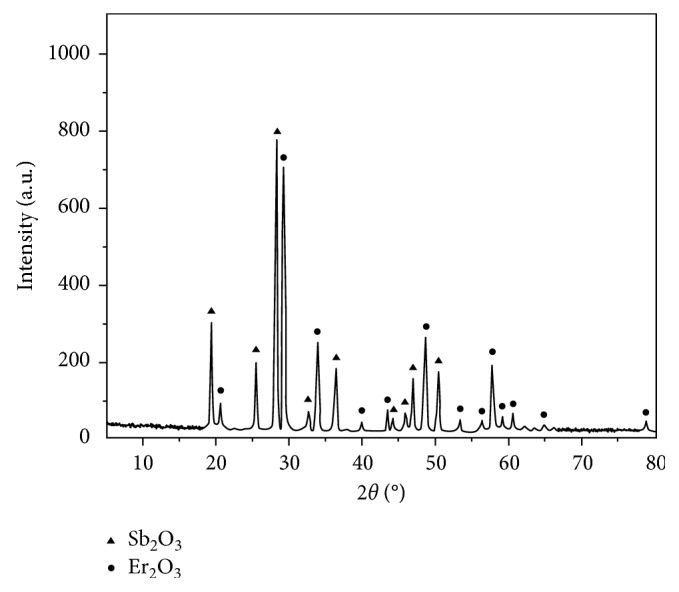
XRD pattern of the pyrolysis residue.

To check the residue, a certain mass of the complex is placed in an alumina crucible and heated in a muffle furnace at 500°C for 2 h. Then the powder X-ray diffraction pattern of the pyrolysis products is recorded. As Figure 7 shows, its characteristic peaks are consistent with the mixture of Sb_2_O_3_ (JCPDS no. 71–0383) and Er_2_O_3_ (JCPDS no. 08–0050). Therefore, the pyrolysis residues must be the mixture of Sb_2_O_3_ and Er_2_O_3_.

### 3.4. Antimicrobial Activity

The culture maintenance and preparation of inoculum were referenced by the literature method [53]. The antimicrobial activities of these compounds were determined qualitatively by agar diffusion method [54]. The inhibition was labeled as the diameter of bacteriostatic circle. A lawn of microorganisms was prepared by pipetting and evenly spreading inoculums (10^6^-10^7^ CFU·cm^−3^) onto agar set in petri dishes, using nutrient agar for the bacteria. Furacilinum was dissolved in DMSO, and penicillin, the title complex, and [Sb(Hedta)]·2H_2_O were dissolved in sterilized water. The Oxford cups were sticked on the previously inoculated agar surface and injected solution of the complex (0.15 mL) under sterile condition. The plates were incubated for 24 h at 37°C. The antimicrobial activity was indicated by the presence of clear inhibition zones around the discs.

Preliminary screening for antimicrobial activities of the complex was performed qualitatively using the disc diffusion assay in Table 4. Each of the compounds was tested three times and the average data were recorded. DMF exhibited no effect on the organisms tested. Furacilinum and penicillin were used as standard drugs, and their activities had been compared with the activities of the title complex. The complex yielded clear inhibition zones around the discs. The results show that the complex has significant antibacterial activities against five tested bacteria, and the antibacterial activities of the sequence are *Escherichia coli*, *Bacillus subtilis*, *Staphylococcus aureus*, *Salmonella typhi*, and *Staphylococcus epidermidis*, respectively. The complex has good antibacterial activity against *Escherichia coli* and *Bacillus subtilis*, and the diameter of inhibition zone of the complex is 26 and 22 mm with the concentration of 1.0 mg·mL^−1^. Meanwhile, the complex shows greater or equal activities against bacteria than the penicillin and furacilinum standard drugs.

## 4. Conclusions

The edta-linked heteronuclear complex [Sb_2_(edta)_2_-*μ*_4_-Er(H_2_O)_4_]NO_3_·4H_2_O was synthesized with erbium nitrate and [Sb(Hedta)]·2H_2_O as the raw materials, due to easy hydrolysis of antimony ion and its weaker coordination than that of erbium with edta^4−^ ion. The complex was characterized by elemental analyses, FTIR spectrum, X-ray diffraction analyses, and thermogravimetry analysis. The crystal structure of the complex belongs to the monoclinic system and space group *Pm* with cell parameters of *a* = 7.3790(10) Å, *b* = 22.116(5) Å, *c* = 10.661(3) Å, *β* = 90.55(2)°, and *Z* = 2. X-ray crystallography analysis reveals that the complex adopts 3-dimensional structures through the weak interactions of antimony and oxygen atoms. The bridging carboxylate-*O*,*O*′ groups of edta^4−^ ions connect with erbium(III) ion and antimony(III) ions. In the complex, the carboxyl oxygen atoms participate in bridging to form diantimony entities and the entities are linked through the carbonyl oxygen atoms to form chains. The metal atoms occupy the space between the chains and are surrounded by the coordinated water molecules, which form hydrogen bonds with the other oxygen atoms of the structure. The complex displays strongly antimicrobial activities on the five tested bacteria.

## Figures and Tables

**Table 1:** Crystallographic data and structure refinement parameters of the title complex.

Empirical formula	C_20_H_40_N_5_O_27_ErSb_2_	*D*_calc_ (g·cm^−3^)	2.278
Formula weight (g·mol^−1^)	1193.33	Absorption coefficient (mm^−1^)	4.043
*T* (K)	289(2)	*F* (000)	1162
Crystal system	Monoclinic	Crystal size (mm^3^)	0.48 × 0.44 × 0.20
Space group	*Pm*	Theta range for data collection (°)	1.84 to 26.00
*a* (Å)	7.3790(10)	Limiting indices	−9 ≤ *h* ≤ 9, −27 ≤ *k* ≤ 27, −13 ≤ *l* ≤ 13
*b* (Å)	22.116(5)	Reflections collected/unique	7847/6826 [*R*(int) = 0.0209]
*c* (Å)	10.661(3)	Goodness-of-fit (GOF) on *F*^2^	1.002
*β* (°)	90.55(2)	Final *R* indices [*I* > 2*σ*(*I*)]	*R*_1_ = 0.0392, *wR*_2_ = 0.1051
*V* (Å^3^)	1739.7(7)	*R* indices (all data)	*R*_1_ = 0.0416, *wR*_2_ = 0.1062
*Z*	2	Largest diff. peak and hole (e·Å^−3^)	1.767 and −1.676

**Table 2:** Selected bond lengths (Å) and angles (°) of the title complex.

Er(1)–O(16)#1	2.285(7)	Er(1)–O(10)	2.290(7)
Er(1)–O(2)	2.290(7)	Er(1)–O(8)#2	2.298(7)
Er(1)–O(20)	2.347(9)	Er(1)–O(17)	2.375(8)
Er(1)–O(19)	2.421(7)	Er(1)–O(18)	2.432(7)
Sb(1)–O(5)	2.127(8)	Sb(1)–O(3)	2.215(8)
Sb(1)–N(1)	2.315(8)	Sb(1)–N(2)	2.332(8)
Sb(1)–O(1)	2.459(7)	Sb(1)–O(7)	2.564(7)
Sb(2)–O(13)	2.124(8)	Sb(2)–O(11)	2.218(9)
Sb(2)–N(3)	2.318(8)	Sb(2)–N(4)	2.322(8)
Sb(2)–O(9)	2.438(7)	Sb(2)–O(15)	2.541(8)
O(16)#1–Er(1)–O(10)	85.6(3)	O(16)#1–Er(1)–O(2)	102.7(3)
O(10)–Er(1)–O(2)	150.5(3)	O(16)#1–Er(1)–O(8)#2	148.0(3)
O(10)–Er(1)–O(8)#2	102.5(3)	O(2)–Er(1)–O(8)#2	85.4(3)
O(16)#1–Er(1)–O(20)	70.8(3)	O(10)–Er(1)–O(20)	80.9(3)
O(2)–Er(1)–O(20)	75.5(3)	O(8)#2–Er(1)–O(20)	140.7(3)
O(16)#1–Er(1)–O(17)	138.7(3)	O(10)–Er(1)–O(17)	74.5(3)
O(2)–Er(1)–O(17)	81.1(3)	O(8)#2–Er(1)–O(17)	72.8(3)
O(20)–Er(1)–O(17)	70.5(3)	O(16)#1–Er(1)–O(19)	78.6(2)
O(10)–Er(1)–O(19)	69.4(3)	O(2)–Er(1)–O(19)	139.7(3)
O(8)#2–Er(1)–O(19)	75.6(3)	O(20)–Er(1)–O(19)	138.7(3)
O(17)–Er(1)–O(19)	124.6(3)	O(16)#1–Er(1)–O(18)	76.3(3)
O(10)–Er(1)–O(18)	139.0(3)	O(2)–Er(1)–O(18)	70.2(3)
O(8)#2–Er(1)–O(18)	77.6(3)	O(20)–Er(1)–O(18)	125.0(3)
O(17)–Er(1)–O(18)	140.0(3)	O(19)–Er(1)–O(18)	71.1(3)
O(5)–Sb(1)–O(3)	146.1(3)	O(5)–Sb(1)–N(1)	79.4(3)
O(3)–Sb(1)–N(1)	73.3(3)	O(5)–Sb(1)–N(2)	75.4(3)
O(3)–Sb(1)–N(2)	79.2(3)	N(1)–Sb(1)–N(2)	77.0(3)
O(5)–Sb(1)–O(1)	82.5(3)	O(3)–Sb(1)–O(1)	105.2(3)
N(1)–Sb(1)–O(1)	68.7(3)	N(2)–Sb(1)–O(1)	142.0(3)
O(5)–Sb(1)–O(7)	99.1(3)	O(3)–Sb(1)–O(7)	91.1(3)
N(1)–Sb(1)–O(7)	142.9(3)	N(2)–Sb(1)–O(7)	67.0(3)
O(1)–Sb(1)–O(7)	148.3(3)	O(13)–Sb(2)–O(11)	146.9(3)
O(13)–Sb(2)–N(3)	81.3(3)	O(11)–Sb(2)–N(3)	72.4(3)
O(13)–Sb(2)–N(4)	75.5(3)	O(11)–Sb(2)–N(4)	79.5(3)
N(3)–Sb(2)–N(4)	77.6(3)	O(13)–Sb(2)–O(9)	80.4(3)
O(11)–Sb(2)–O(9)	107.0(3)	N(3)–Sb(2)–O(9)	68.3(3)
N(4)–Sb(2)–O(9)	140.7(3)	O(13)–Sb(2)–O(15)	96.1(3)
O(11)–Sb(2)–O(15)	93.6(3)	N(3)–Sb(2)–O(15)	143.6(3)

Symmetry transformations used to generate equivalent atoms: #1 *x* − 1/2, −*y*, *z* − 1/2; #2 *x* + 1/2, −*y* + 1, *z* − 1/2; #3 *x* − 1/2, −*y* + 1, *z* + 1/2; #4 *x* + 1/2, −*y*, *z* + 1/2.

**Table 3:** Thermal decomposition data of the title complex.

Reaction	Temperature (°C)	Mass loss (%)	
		*m*_exp_	*m*_theor_
[Sb_2_(edta)_2_-*μ*_4_-Er(H_2_O)_4_]NO_3_·4H_2_O			
↓ −8H_2_O	70–220	12.00	12.08
[Sb_2_(edta)_2_Er]NO_3_			
↓ −NO_2_, −1/4O_2_	220–310	4.28	4.53
[Sb_2_(edta)_2_Er]O_0.5_			
↓ −2(CH_2_)_2_NCH_2_COO, −1/4O_2_	310–360	18.05	17.45
(Sb–Sb)[N(CH_2_COO)_3_]_2_(Er–Er)_0.5_			
↓ −2N(CH_2_)_3_, –2CO	360–430	14.69	14.09
(OSbO–OSbO)(CO)_4_(OErO–OErO)_0.5_			
↓ −4CO, –3/4O_2_	430–510	11.36	11.40
Sb_2_O_3_ + 0.5Er_2_O_3_		39.62^a^	40.45^b^

^a^The experimental mass percent of the residue in the sample; ^b^the calculated mass percent of the residue in the sample.

**Table 4:** Antibacterial activities of the title complex.

Compound	Concentration (mg·mL^−1^)	Inhibition zone diameter (mm)				
		*S*. *aureus*	*E*. *coli*	*S. typhi*	*B. subtilis*	*S. epidermidis*
DMSO	—	—	—	—	—	—
[Sb(Hedta)]·2H_2_O	1.0	14	17	13	14	12
[Sb_2_(edta)_2_-*μ*_4_-Er(H_2_O)_4_]NO_3_·4H_2_O	1.0	17	26	16	22	16
Penicillin	1.0	15	18	17	19	18
Furacilinum	1.0	14	23	19	16	20
